# DESIGN AND VALIDATION OF A BUNDLE FOR ASSESSING GONADAL FUNCTION IN INDIVIDUALS WITH LIVER CIRRHOSIS

**DOI:** 10.1590/S0004-2803.24612026-063

**Published:** 2026-10-05

**Authors:** Nikaelle Ximenes RIOS, Amarílis Cavalcante MONTEIRO, João Guilherme de Mello BATISTA, Elísio Victor Chaves AGUIAR, Renan Martins LOPES, Cristina Micheletto DALLAGO

**Affiliations:** * Universidade Estadual do Ceará, Fortaleza, Ceará, Brasil.

**Keywords:** Liver cirrhosis, hypogonadism, testosterone, bundle, validation study, Cirrose hepática, hipogonadismo, testosterona, pacote de intervenções, estudo de validação

## Abstract

**Background:**

Liver cirrhosis (LC) constitutes the final stage of various chronic liver diseases and represents a serious public health problem. Its pathophysiology involves an inflammatory and fibrotic process that culminates in liver dysfunction and several systemic repercussions, including gonadal dysfunction with consequent hormonal alterations. Despite the clinical importance of this condition, there is a lack of studies that systematically address the assessment of gonadal function in men with LC, justifying the development of specific instruments for this purpose.

**Objective:**

This study aimed to construct and validate a package for assessing gonadal function in men with LC.

**Methods:**

A methodological approach divided into stages of construction and validation of the proposed instrument was used. Initially, an integrative review was conducted using 21 included articles to identify the main clinical and laboratory parameters associated with gonadal dysfunction in men with cirrhosis. These parameters formed the basis for the development of the instrument package, which is structured around three axes: clinical manifestations, laboratory tests, and management of gonadal dysfunction. The instrument underwent content validation by 11 expert judges, selected according to standardized expertise criteria. The evaluation was carried out using a single questionnaire on the Google Forms platform, where judges, based on a Likert scale, assigned scores from 0 to 4 to assess the three domains of the package: i) Objectives; ii) Structure and Presentation; iii) Relevance. Data were analyzed using SPSS 23, employing the Index of Agreement (IA), the Exact Binomial Distribution test (*P*>0.05), and the Intraclass Correlation Coefficient (ICC) to determine reliability.

**Results:**

Validation of the package showed excellent agreement among those evaluated, with a total confidence interval of 98.8% and an ICC of 0.814, demonstrating high reliability of the instrument. All evaluated domains obtained agreement greater than 80%, and the binomial distribution test indicated that the observed agreement proportion was not significantly lower than the predetermined value of 80% (*P*>0.05). The main suggestions from the experts focused on improving the visual and functional aspects of the material, including flowcharts, detailing the procedures, and multidisciplinary integration.

**Conclusion:**

The results confirm the content validity and theoretical consistency of the instrument, establishing it as a useful environmental tool for standardizing the assessment of gonadal function in men with LC.

## HIGHLIGHTS

• Male gonadal dysfunction, particularly hypogonadism, is a common endocrine complication in patients with advanced chronic liver disease, especially cirrhosis, and is associated with metabolic, musculoskeletal, and prognostic impairment.

• The present bundle was developed to provide a standardized, evidence-based framework for the clinical evaluation of gonadal function in cirrhotic patients, addressing the current lack of systematic assessment tools in routine practice.

• Bundle development and validation followed a structured methodological process comprising: a) comprehensive literature review; b) formulation of a clinically applicable assessment bundle; and c) content validation by a panel of expert clinicians.

• The validated bundle demonstrated high clinical relevance and content adequacy, supporting consistent medical decision-making and enabling a practical, reproducible, and effective approach to the evaluation of gonadal dysfunction in patients with liver cirrhosis.

## RESUMO

**Contexto:**

A cirrose hepática constitui o estágio final de diferentes doenças crônicas do fígado e representa um grave problema de saúde pública. Sua fisiopatologia envolve processo inflamatório e fibrótico que culmina em disfunção hepática e múltiplas repercussões sistêmicas, incluindo disfunção gonadal com consequente alterações hormonais. Apesar da importância clínica desta condição, observa-se escassez de estudos que abordem de forma sistematizada a avaliação da função gonadal em homens com cirrose, o que justifica o desenvolvimento de instrumentos específicos para essa finalidade.

**Objetivo:**

Este trabalho teve como objetivo geral construir e validar um bundle para avaliação da função gonadal em homens com cirrose hepática.

**Métodos:**

Utilizou-se um percurso metodológico dividido em etapas de construção e validação do instrumento proposto. Inicialmente, a partir de 21 artigos incluídos, foi realizada a revisão integrativa para a identificação dos principais parâmetros clínicos e laboratoriais associados à disfunção gonadal em homens com cirrose, os quais subsidiaram a construção do bundle em três eixos: manifestações clínicas, exames laboratoriais e condutas frente à disfunção gonadal. O instrumento foi submetido à validação de conteúdo por 11 juízes especialistas, selecionados conforme critérios de expertise padronizados. A avaliação ocorreu por meio da aplicação de um formulário único na plataforma Google Forms, onde os juízes, com base na escala de Likert, atribuíram pontuações de 0 a 4 para avaliar os três domínios do bundle: i) Objetivos; ii) Estrutura e Apresentação; iii) Relevância. Os dados foram analisados no SPSS 23, utilizando-se o Índice de Concordância (IC), o teste Exato de Distribuição Binomial (*p*>0,05) e o Coeficiente de Correlação Intraclasse (CCI) para determinar confiabilidade.

**Resultados:**

A validação do bundle evidenciou excelente concordância entre os avaliadores, com IC total de 98,8% e CCI de 0,814, indicando alta confiabilidade do instrumento. Todos os domínios avaliados obtiveram concordância superior a 80%, e o teste de distribuição binomial indicou que a proporção de concordância observada não foi significativamente inferior ao valor pré-determinado de 80% (*P*>0,05). As principais sugestões dos especialistas concentraram-se na melhoria visual e funcional do material, inclusão de fluxogramas, detalhamento das condutas e integração multiprofissional.

**Conclusão:**

Os resultados confirmam a validade de conteúdo e consistência teórica do instrumento, configurando-o como uma ferramenta potencialmente útil para padronizar a avaliação da função gonadal em homens com cirrose hepática.

## INTRODUCTION

Liver cirrhosis (LC) is characterized as the final stage of a group of chronic liver diseases, making it essential to understand its pathophysiology and complications. According to the World Health Organization (WHO), in 2019 LC was associated with 2.4% of global deaths, being the eleventh most common cause of mortality and accounting for approximately 2 million deaths per year. Persistent hepatitis triggers a continuous fibrotic process which, when advanced, leads to the formation of regenerative nodules and loss of the normal hepatic architecture, thereby characterizing LC. This structural remodeling of the liver progressively results in hepatic dysfunction and failure^1^.

From this perspective, the natural history of LC comprises two stages: the compensated phase, in which the patient remains asymptomatic; and the decompensated phase, in which the patient develops significant complications generally attributed to portal hypertension and hepatic insufficiency, whose outcomes include multiple organ dysfunctions, with special emphasis on gonadal dysfunction^2^.

In LC, there is an increase in the activity of the aromatase enzyme in the liver and adipose tissue, compromising the homeostasis of gonadal hormones, since the liver is responsible for the conversion of androgens into estrogens and for the catabolism of approximately 70% of sex hormones. As a consequence, endocrine dysfunctions occur, such as alterations in the hypothalamic-pituitary-gonadal axis, reduction in total testosterone (TT) production, and increased peripheral conversion of TT into estradiol (E2)^3^.

Although there is relevant evidence of gonadal dysfunction in LC, most studies focus on the male population, which limits the understanding of the particularities of gonadal dysfunction in women with LC. In men, hypogonadism manifests as testicular atrophy, low TT levels, decreased libido, infertility and gynecomastia^4^.

In men with advanced liver disease, low TT levels are associated with anemia, sarcopenia, bone diseases and gynecomastia. Additionally, reduced hormone levels constitute an independent prognostic marker in LC. Testosterone therapy in organic hypogonadism improves muscle mass, bone mineral density, hemoglobin levels, and insulin resistance; however, studies on hormone replacement therapy in these patients remain scarce^5^.

The production of sex hormones in LC varies according to the severity of the disease and factors such as etiology, age, alcohol consumption, and duration of illness. Low TT levels are associated with higher mortality, increased risk of infections, and a greater need for liver transplantation (LTx), regardless of similar Model for End-Stage Liver Disease (MELD) scores. Despite this, there is a lack of studies that comprehensively evaluate reproductive hormones and their correlation with prognostic scores such as MELD and Child-Turcotte-Pugh^3^ ^,^ ^6^.

In LC, alterations in sex hormones lead to relevant clinical repercussions, such as sarcopenia, which negatively impacts quality of life and prognosis, including after LTx. Nevertheless, there are few studies on gonadal function, especially interventional ones. In this context, according to the Institute for Healthcare Improvement, the importance of using a bundle as an innovative tool for evaluating gonadal function in patients with LC is highlighted, particularly in advanced disease and in LTx, since the integrated application of a set of specific care measures promotes significant improvements in healthcare delivery^7^.

Bundles consist of the systematic and combined application of three to five evidence-based measures that, when executed completely and properly, improve patient outcomes, enhance the quality of care, and reduce morbidity and mortality^8^. Therefore, the relevance of this study lies in the development of a structured bundle based on an extensive literature review, with the aim of facilitating the practical and effective investigation of gonadal dysfunction in patients with LC.

## METHODS

### Studies characteristics

The present study is a methodological research targeting the construction and validation of a bundle to approach gonadal function in men with LC. The period of development and validation of the bundle was from June 2024 to December 2025. The validation of the bundle included judges who were specialists in gastroenterology and hepatology and were selected based on criteria that will be discussed in the following sections. The project was submitted to the evaluation of the Ethics in Research Committee through a register on “Plataforma Brasil”, receiving approval according to substantiated report no. 6,850,732.

### Research steps

The execution of the proposed study with the construction and validation of the bundle was developed based on the following steps: 1) bibliographic search; 2) development of the bundle; 3) validation of the bundle’s content by expert judges.

### Integrative review

The bibliographic search was established based on an integrative review about the evidence related to gonadal function in patients with LC present in Medline/Pubmed, Lilacs e Embase scientific data bases. The research question was defined according to the PICo (P = Population, I = Phenomena of interest and Co = context) strategy.

In this research it was established that the element “P” are the male patients with liver cirrhosis, the “I” the construction of the bundle and the “Co” the clinical and laboratory parameters of assessment of the gonadal male function on cirrhotic patients.

The terms used in the searching process were combined with each other using the Boolean operator “AND”, delimited with the Health Sciences Descriptors (DeCS), Medical Subject Headings (MeSH) and EMTREE, in accordance with the PICo strategy.

The selection of material followed these inclusion criteria: original scientific articles, fully available online; in Portuguese, English, or Spanish; thematic content related to alterations in gonadal function in patients with LC; time frame: 2014 to 2024. The selection and organization of studies was directed and executed according to the PRISMA^9^ flowchart guidelines.

### Elaboration and validation of the bundle

The evidence found in the integrative review supported the development of the bundle’s content, whose aimed target audience is composed of endocrinologists and gastroenterologists, seeking the development of more effective strategies for screening gonadal dysfunction in patients with LC.

Initially, the evaluation of gonadal function in both sexes was proposed. However, as observed in the results of the integrative review, there is a shortage of studies on this topic in women, which impaired the construction of a specific care assessment that also encompassed the female sex. Considering that the literature presents greater robustness of data, clinical and laboratory parameters related to the male sex, it was decided to limit the investigation to this population.

The bundle was structured to encompass 3 axes focused on clinical assessment and management, which were clinical manifestations, laboratory tests, and conduct. It aimed to address the following needs of medical practice: to guide the management of these patients and potential complications; to direct the tests to be requested in the investigation of gonadal dysfunction, providing guidance on appropriate treatment for different situations of regarding alterations in the gonads (medication or conservative management); and to instruct on how to follow up the patients.

The validation phase included expert judges who were selected based on criteria that assumed: having clinical experience with patients with LC, publishing and researching on the topic, being an expert in the conceptual framework involved, and/or having methodological knowledge about the construction of instruments applied in the health field, so that they were able to adequately assess the representativeness or relevance of the content.

For this study, medical professionals (gastroenterologists and hepatologists) with experience in monitoring patients with LC, including those in pre-hepatic transplant evaluation and post-hepatic transplant follow-up, were invited to form the panel of judges. The selection of judges was carried out through analysis of the curriculum available on the Lattes Platform, to ensure the necessary expertise of the evaluator for the validation of the instrument.

The judges were selected using the scoring system proposed by Joventino^10^. Judges had to meet the minimum required criteria (5 points) to be invited to participate in the study. After identifying potential participants, a single link to the Google Forms platform was sent.

The judges had to evaluate each item of the construct using a Likert scale, assigning a score from 1 to 4, the interpretation of which varies according to the domain analyzed. For domains 1 and 2, the score corresponds to: 1) inadequate; 2) partially adequate; 3) adequate; and 4) fully adequate. For domain 3, the following criteria were adopted: 1) not relevant or not representative; 2) item that needs significant revision to be representative; 3) item that needs minor revision to be representative; and 4) item that is relevant or representative.

The evaluation was divided into three domains: i) Objectives; ii) Structure and Presentation; iii) Relevance. On this scale, respondents positioned themselves according to a measure of agreement assigned to each item, in order to infer the measure of the construct, as detailed in the following section.

### Statistical analysis

The data from the expert judges were imported from Google Forms and saved as a spreadsheet in Microsoft Excel. Descriptive and inferential statistical analyses were performed using the Statistical Package for the Social Sciences (IBM SPSS), version 23.

In characterizing the judges, absolute and relative frequencies were calculated for categorical variables; for numerical variables, normality was analyzed using the Shapiro-Wilk test, followed by the calculation of measures of central tendency and dispersion (minimum and maximum values, mean, and standard deviation).

The veracity of the evidence was assessed using the Index of Agreement (IA), which measures the proportion of agreement among experts regarding the items and general aspects of the instrument, allowing individual and collective analysis of the items. Based on the percentage estimate of the IA formula, a result ≥90% means that the domains are adequate; if IA ≤90%, it indicates the need to modify the domain^11^ ^,^ ^12^ (Figure 1).

**FIGURE 1:**
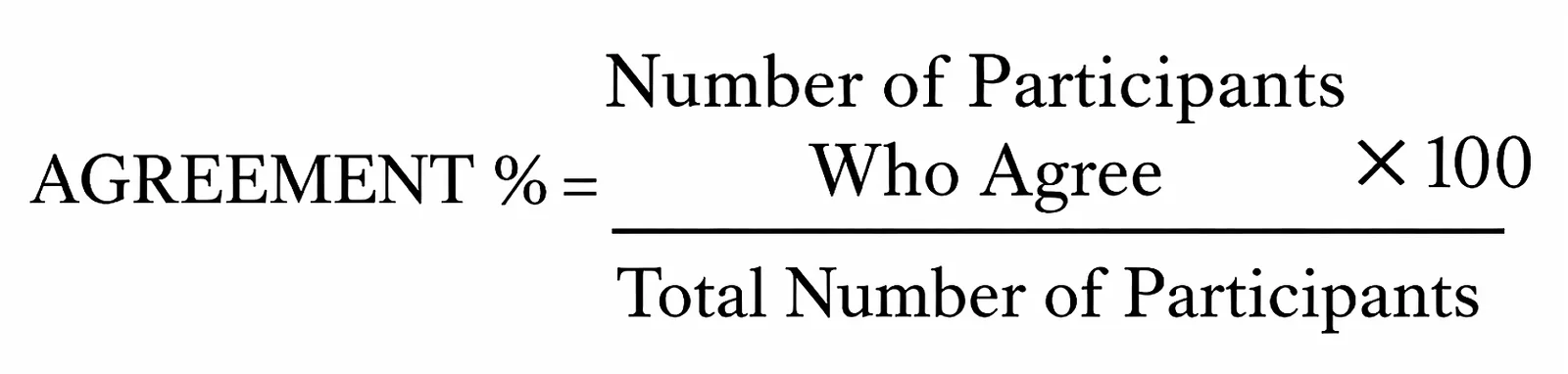
Calculation of the Index of Agreement. Source: adapted from Coluci, Alexandre and Milani^13^.

The cutoff point proposed by Polit, Beck, and Owen was used, in which: ≥0.78 is considered excellent; between 0.60 and 0.77 indicates a need for adjustment; and <0.60 is classified as poor and the material should be reviewed from the perspective of the poorly scored item^14^.

Statistical tests were also performed to evaluate the distribution of scores assigned by judges regarding disagreement and reliability. In the first case, the Exact Binomial Distribution test was calculated to verify whether the proportion of evaluators was statistically equal to or greater than the predetermined value, with a significance level of 5% (*P*>0.05) and a proportion of 0.80 for agreement. In the test, if the *P*-value for each item is less than 0.05, it indicates significant disagreement among the judges in the scoring^15^.

The reliability of the score distribution was explored using the Intraclass Correlation Coefficient (ICC) and its 95% intervals, through a two-way mixed-effects model and definition of consistency relation. Koo and Li propose the following reference values: poor reliability (<0.50), moderate (between 0.50 and 0.75), good (0.75 to 0.90) and excellent (>0.90)^16^.

The evaluation was conducted by 11 judges, selected based on the criteria already described. It was stipulated that, if the minimum IA was not reached, the bundle would be reformulated and sent to the expert committee for further evaluation, so that the experts’ suggestions could be analyzed and considered for adjustments to the bundle after its validation. However, the minimum IA was reached in all items, making further evaluation unnecessary.

## RESULTS

The following flowchart details the methodology used to select information during the integrative review (Figure 2).

**FIGURE 2:**
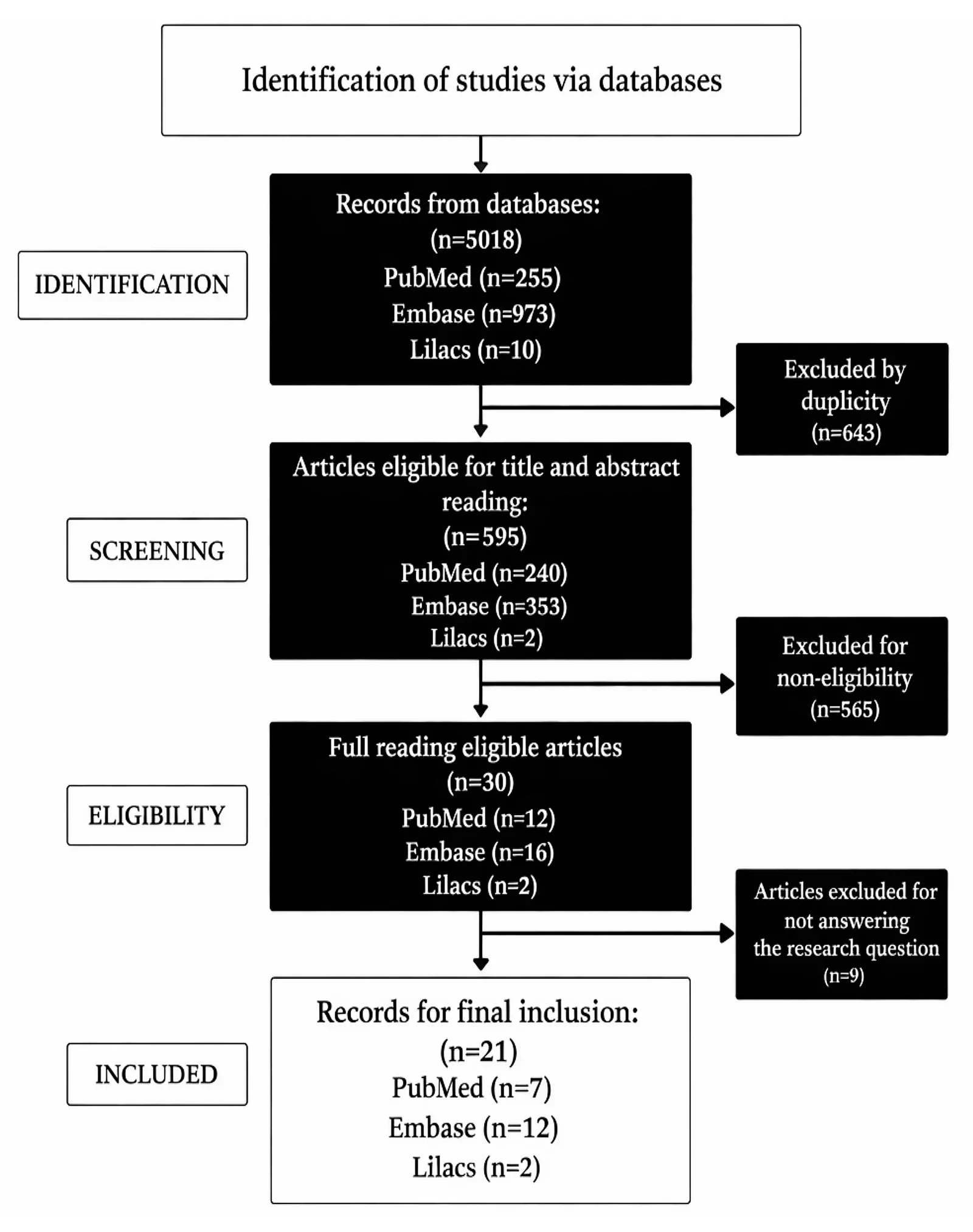
The process of selecting articles from databases. Source: made by the authors (2026).

Among the years of the 21 studies that make up the integrative review, the period encompasses 2014 to 2024, with prevalence in descending order of frequency: 2022 (n=4)^32^ ^-^ ^35^, 2016 (n=3)^18^ ^,^ ^19^ ^,^ ^21^, 2021 (n=3)^29^ ^-^ ^31^, 2019 (n=3)^25^ ^-^ ^27^, 2018 (n=2)^22^ ^,^ ^24^, 2014 (n=2)^17^ ^,^ ^23^, 2020 (n=1)^28^, 2023 (n=1)^36^, 2015 (n=1)^20^ and 2024 (n=1)^37^.

Regarding the research locations, the predominance followed this order: Australia (n=4)^18^ ^-^ ^21^; China (n=4)^26^ ^,^ ^29^ ^,^ ^30^ ^,^ ^35^; India (n=3)^32^ ^,^ ^36^ ^,^ ^37^; Brazil (n=2)^17^ ^,^ ^23^; United States (n=2)^31^ ^,^ ^34^; Canada (n=2)^22^ ^,^ ^32^; Taiwan (n=1)^24^; Austria (n=1)^25^; Japan (n=1)^27^; Belgium (n=1)^28^; Egypt (n=1)^33^.

Furthermore, prospective cohort studies (N=10) (level of evidence IV) were the most prevalent among those included, followed by cross-sectional studies (N=7) (level of evidence V), retrospective cohort studies (N=3) (level of evidence IV), and randomized clinical trials (N=1) (level of evidence II) (Table 1).

**TABLE 1:** Characterization of the included studies.

Code	Author/Year	Country	Objective	Study Design	Main Results	LOE
R17	Zacharias et al. (2014)	Brazil	To evaluate the influence of end-stage liver disease and orthotopic liver transplantation (OLT) on pituitary function and hormonal metabolism by measuring serum levels of FSH, LH, E2, and PRL before and after liver transplantation.	Prospective cohort	• Pre-LTx: normal/inappropriately low FSH; increased E2 and PRL.	IV
					• Post-LTx: increased FSH/LH; E2 and PRL normalized.	
					• MELD showed no influence.	
					• Greater E2 normalization in severe cirrhosis.	
R18	Sinclair et al. (2016)	Australia	To evaluate the association between sarcopenia and low serum testosterone with increased mortality in men with cirrhosis.	Retrospective cohort	• Low TT (179 ng/dL) associated with sarcopenia and MELD.	IV
					• Low TT was an independent predictor of mortality.	
R19	Sinclair et al. (2016)	Australia	To evaluate the impact of testosterone on mortality in men awaiting liver transplantation.	Prospective cohort	• Median TT: 501 ng/dL;	IV
					• 23.9% TT deficient;	
					• 32.8% low FT;	
					• 28.2% ↑ LH indicating pituitary response to low TT.	
R20	Sinclair et al. (2015)	Australia	To perform a comprehensive analysis of androgen status and circulating estrogens in men with cirrhosis.	Cross-sectional study	• Estrone/E2 ↑ in cirrhotic patients.	V
					• Estrone correlated with MELD (↑) and sodium/Hb (↓).	
					‘• TT inversely correlated with MELD and positively with hand-grip strength, physical activity, Hb, and Na.	
					• DHT followed TT pattern.	
R21	Sinclair et al. (2016)	Australia	To investigate whether testosterone therapy in cirrhosis improves muscle mass and other outcomes.	Randomized clinical trial	• Testosterone therapy increased lean mass, bone mass, and hemoglobin;	II
					‘• Reduced fat mass and HbA1c.	
‘					• No significant mortality reduction and no increase in adverse events.	
R22	Moctezuma-Velázquez et al. (2018)	Canada	To investigate whether sarcopenia is associated with low testosterone levels in patients with cirrhosis.	Retrospective cohort	‘• Reduced TT in sarcopenic patients.	IV
					• Hypotestosteronemia associated with sarcopenia independent of MELD and etiology.	
R23	Nitsche et al. (2014)	Brazil	‘	Prospective cohort	‘• Post-LTx: increased TT and FT.	IV
					• SHBG decreased and normalized in patients with MELD ≤18.	
R24	Chiang et al. (2018)	Taiwan	To evaluate the predictive role of MELD score for changes in testosterone levels after living donor liver transplantation (LDLT) and its effects on SHBG, FAI, and erectile function.	Prospective cohort	• LDLT increased FAI at 1, 3, and 6 months.	IV
					• Improvement in erectile dysfunction with higher IIEF-5 scores.	
R25	Paternostro et al. (2019)	Austria	To evaluate sexual hormone status in relation to hemodynamic abnormalities and its impact on hepatic decompensation and survival.	Prospective cohort	• TT and FT decreased with advanced Child-Pugh.	IV
					• PRL increased. Lower TT and FT in severe portal hypertension and sarcopenia.	
					• Low TT associated with higher risk of decompensation and death independent of MELD or Child-Pugh.	
R26	Chan et al. (2019)	China	To determine the incidence of erectile dysfunction before and after liver transplantation and its correlation with changes in sex hormone levels.	Prospective cohort	• Post-LTx: decreased and normalized E2 and PRL.	IV
					• Hormonal improvement was not accompanied by improvement in erectile dysfunction.	
R27	Fujihara et al. (2019)	Japan	To clarify the relationship between serum testosterone or SHBG and the fibrosis index (FIB-4).	Cross-sectional study	• Increased SHBG and decreased FT associated with greater fibrosis (FIB-4 ≥1.3).	V
					• FT <7.62 ng/dL and SHBG ≥49.8 nmol/L were major predictors of severity.	
Code	Author/Year	Country	Objective	Study Design	Main Results	LOE
R28	Van de Velde et al. (2020)	Belgium	To investigate whether severity and histological components of NAFLD are associated with sex steroid levels in obese men.	Cross-sectional study	• NASH is associated with lower FT and higher E2/FT ratio.	V
					• Steatosis associated with lower TT and FT.	
					• Obese patients with NASH had even lower FT levels.	
R29	Sun et al. (2021)	China	To explore the effect of testosterone and E2 on the occurrence and prognosis of HBV-ACLF.	Prospective cohort	• HBV-ACLF is associated with increased E2 and reduced TT.	IV
					• TT predicted mortality and E2 predicted ACLF occurrence.	
					• TATIM prognostic model showed good predictive accuracy.	
R30	Huang et al. (2021)	China	To investigate the association between testosterone levels, clinical stages, severity scores, and outcomes in HBV-ACLF.	Prospective cohort	• Decreased TT, FTI, DHEA-S, and cortisol; increased androstenedione.	IV
					• Reduced TT, SHBG, and FT associated with higher MELD and ACLF.	
					• TT <142.39 ng/dL predicted 90-day mortality.	
R31	Sarkar et al. (2021)	United States	To evaluate whether testosterone influences NAFLD severity in women.	Cross-sectional study	• Young women: ↑ FT associated with NAFLD, fibrosis, and higher abdominal adiposity.	V
					• FT associated with disease severity.	
R32	Singh et al. (2022)	India / Canada	To describe hormonal variation and osteodystrophy among frail and non-frail patients with cirrhosis.	Prospective cohort	• Frail group: ↓ TT, PTH, vitamin D3, and cortisol.	IV
					• Higher prevalence of osteodystrophy.	
					• Bone mineral density correlated with TT, vitamin D3, PTH, and cortisol.	
R33	Hassan et al. (2022)	Egypt	To evaluate the effect of sarcopenia on survival and complications after liver transplantation.	Prospective cohort	• Sarcopenia present in 44.3% pre-LTx; decreased to 30.4% post-LTx.	IV
					• Pre-LTx sarcopenia and age were independent predictors of post-LTx mortality.	
R34	Jain et al. (2022)	United States	To define postoperative functional sarcopenia after LTx according to TT level and evaluate short-term testosterone replacement therapy with active exercise.	Retrospective cohort	• Very low TT (28.3 ng/dL). • Patients treated with testosterone cypionate. Survival at 1 and 5 years: 93.8% and 87.5%. Five patients were sarcopenic and six had low muscle quality.	IV
						
R35	Zhang et al. (2022)	China	To investigate whether testosterone is associated with NAFLD progression in men with type 2 diabetes.	Cross-sectional study	• TT quartiles inversely associated with NAFLD inflammation.	V
					• ↑ TT associated with fibrotic progression. Effect modified by age, diabetes, and dyslipidemia.	
R36	Vaishnav et al. (2023)	India	To study sex hormones in men with cirrhosis and determine their correlation with prognostic scores.	Cross-sectional study	• Cirrhotics: ↓ FT, ↑ E2, ↓ FT:E2 ratio vs controls. LH/FSH/PRL unchanged.	V
					• ↓ FT is associated with age, alcoholism, duration of cirrhosis, reduced libido, and ascites.	
					• FT correlated with Child-Pugh but not MELD-Na.	
R37	Pal et al. (2024)	India	To evaluate clinical, biochemical, and hormonal abnormalities in patients with different stages of alcoholic liver disease.	Cross-sectional study	• Steatohepatitis, alcoholic hepatitis, and cirrhosis: L TT (greater in cirrhosis).	V
					• ↑ E2 (57-70%).	
					• ↑ PRL (53-63%).	
					• Hormonal alterations accompanied by anemia, hypoalbuminemia, and laboratory abnormalities.	

TT: total testosterone. FT: free testosterone. E2: estradiol. PRL: prolactina. SHBG: sex hormone-binding globulin. MELD: Model for End-Stage Liver Disease. ACLF: acute-on-chronic liver failure. NAFLD: nonalcoholic fatty liver disease. NASH: nonalcoholic steatohepatitis. LTx: liver transplantation. FAI: free androgen index. DHEA-S: dehydroepiandrosterone sulfate. PTH: parathyroid hormone. Hb: hemoglobin. Na: sodium. LDLT: living donor liver transplantation. IIEF-5: International Index of Erectile Function-5. FIB-4: Fibrosis-4 Index. HbA1c: glycated hemoglobin. BMD: bone mineral density. ALD: alcoholic liver disease. LOE: level of evidence. TATIM: the novel prognostic model involving testosterone. Source: made by the authors (2026).

### Elaboration of the bundle

After analyzing these studies, it was observed that, in patients with LC, the clinical history and physical examination are directly influenced by gonadal dysfunction, particularly in laboratory tests. In this context, the construction of the bundle respects three major axes described in the methodological section, which will be discussed in detail.

The first axis, clinical manifestations, is composed of the predominant clinical history of the profile of these patients under study, highlighting finds of gynecomastia, testicular atrophy, sarcopenia, reduction or change in hair distribution, gynecoid-pattern adiposity, decreased libido, erectile dysfunction, lower fertility rate, and altered voice timbre. All these signs and symptoms can be grouped into the first axis of clinical manifestations^3^ ^,^ ^22^ ^,^ ^31^.

Following this search for the aforementioned components of gonadal dysfunction, healthcare professionals can investigate the hypothesis of gonadal dysfunction, given its poor prognosis for patients with LC during the patient’s evolution. Due to this severity, serum levels of TT^18^ ^,^ ^24^ ^,^ ^27^ ^,^ ^34^, free testosterone (FT)^3^ ^,^ ^27^ ^,^ ^31^, sex hormone-binding globulin (SHBG)^29^, follicle-stimulant hormone (FSH) and luteinizing hormone (LH)^35^, E2^17^ ^,^ ^29^ and the FT/TT ratio^31^, may be associated with the severity of LC, favoring the presence of sarcopenia, osteodystrophy, and other complications observed in this patient profile.

However, despite the understanding of gonadal dysfunction in LC, there is no consensus in the literature on how to evaluate these patients in medical practice. This situation in the literature contributes to divergent results, especially in the management of these dysfunctions, given the shortage of studies that recommend hormone replacement therapy with statistical significance as a therapeutic alternative.

In summary, due to the limited availability of resources to guide the management and follow-up of this patient group, this bundle unified the most relevant components listed in the literature to offer a structured clinical assessment tool capable of guiding decisions for healthcare professionals through the anamnesis and physical examination of patients with LC, as shown in Figure 3.

**FIGURE 3:**
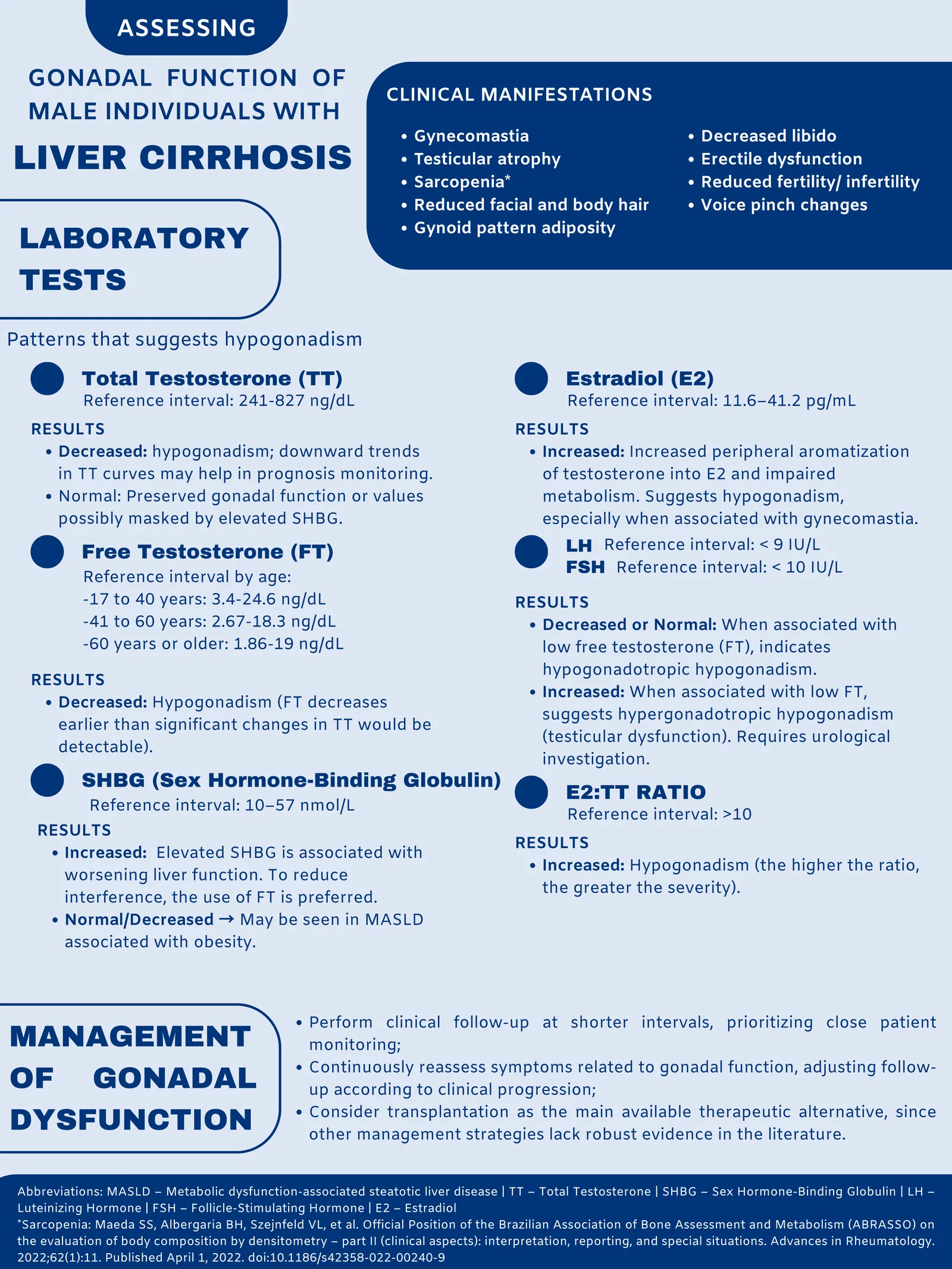
Visual project of the bundle. Source: made by the authors (2026).

### Evidence of content validity and clinical applicability

The characteristics of the judges are described in Table 2, with the majority being female (n=6; 54.5%), aged 37 to 52 years (44.8±4.4), specialists in hepatology (n=8; 72.7%) and gastroenterology (n=6; 54.5%), working in the area for 6 to 23 years (13.5±5.3) and holding a master’s degree (n=6; 54.5%).

**TABLE 2:** Characteristics of the Judges.

Variables	n = 11	%	Other statistics
Social			
Sex			
Female	06	54.5	
Male	05	45.5	
Age (years)			Min.= 37; Max.= 52; Mean = 44.8; SD = 4.4
Professional			
Training (specialty)			
Gastroenterology	06	54.5	
Hepatology	08	72.7	
Years of practice in the specialty			Min.= 6; Max.= 23; Mean = 13.5; SD = 5.3
Academic degree			
Specialization	03	27.3	
Master’s degree	06	54.5	
Doctorate	02	18.2	
Field of study			
Cirrhosis/Liver fibrosis	02	18.2	
Gastroenterology	02	18.2	
Hepatology	03	27.3	
Liver transplantation	06	54.5	
Hepatocellular carcinoma	01	9.1	
Works in the care of patients with gonadal dysfunction? (yes)	04	36.6	
Works in the care of patients with liver cirrhosis? (yes)	11	100.0	
Years of experience caring for patients with liver cirrhosis			Min.= 8; Max.= 21; Mean = 13.3; SD = 5.1
Works in the care of patients in the pre-liver transplantation period? (yes)	11	100.0	
Years of experience caring for patients in the pre-liver transplantation period			Min.= 8; Max.= 21; Mean = 12.6; SD = 4.4
Participates in research groups or projects in the areas of gonadal dysfunction, cirrhosis, and/or liver transplantation? (yes)	08	72.7	
Years of participation in research groups or projects in the areas of gonadal dysfunction, cirrhosis, and/or liver transplantation			Min.= 6; Max.= 21; Mean = 11.5; SD = 5.3
Has publications in the areas of gonadal dysfunction, cirrhosis, and/or liver transplantation? (yes)	08	72.7	

n: absolute frequency. %: relative frequency. Min.: minimum value. Max.: maximum value. SD: standard deviation. a: variable allows more than one response option.Source: made by the authors (2026).

LTx is the most studied area among the evaluators (6; 54.5%); four judges (36.6%) confirm attending to patients with gonadal dysfunction; all evaluators (100%) have been assisting patients with LC for a period of 8 to 21 years of experience (13.3±5.1); furthermore, all (100%) participated in the follow-up prior to LTx with the same variation in experience between 8 and 21 years (12.6±4.4); eight of these evaluators (72.7%) stated that they participate in research in the areas of gonadal dysfunction, LC and/or LTx with experience between 6 and 21 years (11.5±5.3) and eight evaluators (72.7%) had publications in the mentioned areas.

The validation of the bundle’s content can be seen in Table 3. Regarding the Objective domain, the Index of Agreement of the Item (IA-I) ranged from 90.9% to 100%. Concerning the items in this section, the judges considered them consistent with the needs of medical practice (IA-I=100%; *P*=0.054) and useful for guiding the assessment of gonadal function (IA-I=90.9%; *P*=0.383); in addition to encouraging the adoption of new patient care practices (IA-I=100%; *P*=0.383), suitable for circulation in the scientific community (IA-I=100%; *P*=0.383) by agreeing with the objectives of institutions that provide care to men with LC (IA-I=100%; *P*=0.161). The IA-I for this domain was 98.2, with no significant disagreement among the judges in the distribution of scores.

**TABLE 3:** Evaluation of the Bundle by Experts.

Domains	Agreement			I-CVI (%)	***P*-value**
	D n (%)	A n (%)	TA n (%)		
**Objective**				**98.2**	
1. The information/content is consistent with the needs of physicians who conduct clinical follow-up and indicate liver transplantation for cirrhotic men.	-	05 (45.5)	06 (54.5)	100.0	0.054ª
2. The information/content assists specialist physicians in evaluating gonadal function in men with liver cirrhosis.	01 (9.1)	02 (18.2)	08 (72.7)	90.9	0.383ª
3. Encourages and/or promotes the adoption of new practices, including changes in professional behaviors and attitudes toward the care of patients with cirrhosis.	-	02 (18.2)	09 (81.8)	100.0	0.383ª
4. It can circulate in the scientific community of the field.	-	03 (27.3)	08 (72.7)	100.0	0.383ª
5. Meets the objectives of institutions that provide care to men with liver cirrhosis.	-	04 (36.4)	07 (63.6)	100.0	0.161ª
**Structure and Presentation**				**98.2**	
6. The bundle is appropriate for physicians who conduct clinical follow-up and indicate liver transplantation.	-	02 (18.2)	09 (81.8)	100.0	0.617
7. The messages are presented clearly and objectively.	-	05 (45.5)	06 (54.5)	100.0	0.054ª
8. The information presented is scientifically correct.	-	03 (27.3)	08 (72.7)	100.0	0.383ª
9. The material is appropriate to the scientific level of physicians who conduct clinical follow-up and indicate liver transplantation.	-	03 (27.3)	08 (72.7)	100.0	0.383ª
10. There is a logical sequence of the proposed content.	02 (18.2)	05 (45.5)	04 (36.4)	81.8	0.383ª
11. The information is well structured in terms of grammar and spelling.	-	03 (27.3)	08 (72.7)	100.0	0.383ª
12. The writing style corresponds to the scientific level of physicians who conduct clinical follow-up and indicate liver transplantation.	-	05 (45.5)	06 (54.5)	100.0	0.054ª
13. The font size of the title and topics is appropriate.	-	02 (18.2)	09 (81.8)	100.0	0.617
14. The material (paper/printing) is appropriate.	-	03 (27.3)	08 (72.7)	100.0	0.383ª
15. The number of items is adequate.	-	03 (27.3)	08 (72.7)	100.0	0.383ª
				**100.0**	
16. The theme portrays key aspects that should be reinforced.	-	01 (9.1)	10 (90.9)	100.0	0.322
17. The bundle allows generalization and transfer of learning to different contexts.	-	05 (45.5)	06 (54.5)	100.0	0.054ª
18. The bundle proposes the construction of knowledge.	-	01 (9.1)	10 (90.9)	100.0	0.322
19. The bundle addresses the necessary care to increase the level of knowledge of physicians who conduct clinical follow-up and indicate liver transplantation.	-	02 (18.2)	09 (81.8)	100.0	0.617
20. The bundle is suitable for use by any specialist physician experienced in the care of patients with cirrhosis.	-	04 (36.4)	07 (63.6)	100.0	0.161ª
total ICI	-	-	-	98.8	-
ICC (IC95%)	0.814 (0.606 - 0.940)			-	-

D: disagree. A: agree. TA: totally agree. IA-I: Index of Agreement of the Item. p-value: Exact Binomial Test. ICC (95% IA): Intraclass Correlation Coefficient with 95% confidence interval.

In the Structure and Presentation domain, the judges considered the bundle appropriate (IA=100%; *P*=0.617), with clear and objective language (IA=100%; *P*=0.054), scientifically correct (IA=100%; *P*=0.383) and suitable for the scientific level of medicine (IA=100%; *P*=0.383); content with logical sequence (IA=100%; *P*=0.383) and structured in terms of agreement and spelling (IA=100%; *P*=0.383); with adequate writing (IA=100%; *P*=0.054). The font size (IA=100%; *P*=0.617) and number of items (IA=100%; *P*=0.383) are adequate and the material is appropriate (IA=100%; *P*=0.383). The domain presented items ranging from 81.8% to 100%, with a 98.2% confidence interval and no significant disagreement.

Next, the Relevance domain presented the highest score (100%), as all items obtained the maximum score. The bundle presents a theme that portrays key aspects (IA=100%; *P*=0.322), allows for generalization and transfer of learning (IA=100%; *P*=0.054), proposes knowledge construction (IA=100%; *P*=0.322), addresses necessary care (IAI=100%; *P*=0.617), and is suitable for use by any specialist physician with experience in assisting patients with LC (IA=100%; *P*=0.161).

Considering all these aspects, a bundle with a total confidence interval of 98.8% was obtained, demonstrating its theoretical excellence, with no significant disagreement among the judges in the distribution of scores for all items, and with good reliability (ICC=0.814), as attested by its confidence intervals. Table 4 lists the judges’ considerations regarding the bundle.

**TABLE 4:** Judges suggestion’s.

Judge	Suggestions
J3	Insert a flowchart showing the order of requesting tests; prioritize a design suitable for smartphone screen viewing.
J4	Include doses, contraindications, and follow-up procedures.
J6	In the section “management of gonadal dysfunction,” cite evidence regarding which tests are necessary and validated, listing the recommended testing frequency; stratify whether there are cirrhotic patients who may receive treatment for hypogonadism.
J8	Add: in case of abnormalities, refer the patient to the endocrinology outpatient clinic for joint follow-up while awaiting liver transplantation; associate with nutritional assessment and physical exercise; cite possible complementary imaging methods such as testicular ultrasound and bone densitometry.
J9	Include therapies with robust evidence for the treatment of hypogonadism in cirrhotic patients; clarify whether all tests should be requested at once or if there is a hierarchical sequence for ordering hormonal markers of hypogonadism; address management in cases of gonadal dysfunction.
J10	Include a flowchart suggesting a sequence of tests and management actions to optimize evaluation.

Source: made by the authors (2026).

After evaluating the judges’ considerations for improving the bundle, it was found that the main recommendations include improving the visual and functional components, detailing clinical information, and integrating a multidisciplinary approach.

## DISCUSSION

End-stage liver disease is strongly associated with gonadal dysfunction, which contributes to worsening clinical prognosis. The findings of this review indicate that up to 90% of men with liver cirrhosis exhibit significant reductions in TT levels, with a direct correlation between the severity of liver disease and the degree of hormonal dysregulation^5^ ^,^ ^17^ ^,^ ^18^ ^,^ ^20^.

Clinically, suspicion of hypogonadism in men with liver cirrhosis should be investigated during clinical history-taking, focusing on symptoms such as decreased libido, erectile dysfunction, fatigue, gynecomastia, testicular atrophy, redistribution of body hair, and other signs of feminization. These clinical features may allow for early recognition of the condition, even before laboratory abnormalities become apparent^3^ ^,^ ^17^ ^,^ ^20^ ^,^ ^22^.

In this context, low TT levels in men have been consistently associated with adverse outcomes and increased mortality, according to meta-analytic evidence. Although the diagnostic thresholds used across studies vary, robust data indicate that TT concentrations between 153-250 ng/dL (5.3-8.7 nmol/L) are associated with a higher risk of all-cause mortality, and in some analyses, with increased cardiovascular- and cancer-related mortality. In metabolic dysfunction-associated steatotic liver disease (MASLD), TT levels may be reduced due to decreased SHBG levels and testicular dysfunction related to insulin resistance^34^.

Regarding the assessment of FT levels, reduced concentrations confirm hypogonadism and may provide greater diagnostic accuracy than TT alone. In patients with MASLD and obesity, FT levels may remain within the normal range or be only mildly reduced due to dysregulation of the hypothalamic-pituitary-gonadal axis and hepatic dysfunction. A more pronounced reduction is typically observed in advanced stages of the disease. Elevated levels of SHBG generally indicate advanced LC in the absence of acute hepatic failure and may change earlier than TT levels. Conversely, reduced SHBG concentrations are commonly associated with insulin resistance and are frequently observed in the coexistence of MASLD and obesity^3^ ^,^ ^29^.

Reduced levels of both TT and FT are inversely correlated with the severity of liver cirrhosis, as assessed by the MELD score, and are associated with lower serum sodium levels, reduced handgrip strength, and decreased hemoglobin levels^23^ ^,^ ^29^. Conversely, increased prolactin and estradiol levels are observed in advanced stages of liver disease, reflecting dysregulation of the hypothalamic-pituitary-gonadal axis and alterations in peripheral hormone metabolism^3^ ^,^ ^17^ ^,^ ^19^.

This finding is further supported by studies demonstrating significantly higher mortality among men with a MELD score ≤16 and reduced TT levels, highlighting the prognostic value of this biomarker. Beyond its anabolic effects, testosterone also exerts immunomodulatory and anti-inflammatory actions. Therefore, testosterone deficiency may plausibly contribute to insulin resistance, impaired hematopoiesis, and systemic inflammation, factors that may accelerate the clinical progression of liver cirrhosis.

There is considerable divergence among studies regarding the influence of the MELD score on hormonal alterations. Some investigations report an effect of MELD primarily on E2 levels, without significant impact on FSH, LH, or prolactin, whereas others have not identified a direct correlation between MELD and TT or FT levels. These inconsistencies likely reflect methodological differences, heterogeneity among the studied populations, and variability in the stages of liver disease across cohorts.

Another finding frequently reported in the analyzed studies is the association between hypogonadism and sarcopenia. Low TT levels have been correlated with reduced muscle mass, with negative implications for mortality both before and after LTx^21^ ^,^ ^23^.

Although the association between sarcopenia and TT appears consistent in men, this relationship has not been observed in women. This finding may be explained by the smaller sample size and the lower prevalence of female hypogonadism, as only 16% of the evaluated women exhibited reduced TT levels^30^.

The lack of statistical significance in this subgroup may also reflect physiological and hormonal particularities of the female sex, highlighting the scarcity of data in this population and supporting the focus of the present study on male individuals. This limitation has been emphasized in several studies that point to the paucity of robust research on gonadal dysfunction in women with LC^3^ ^,^ ^21^ ^,^ ^23^ ^,^ ^30^.

The available literature demonstrates a scarcity of data on women with LC, with most studies predominantly involving male participants. This imbalance limits the understanding of sex-related differences in gonadal dysfunction and supports the focus on male populations, in whom the prognostic value of androgens is more clearly established.

In patients with hepatitis B-related acute-on-chronic liver failure (ACLF), TT has been identified as an independent predictor of short-term mortality, whereas estradiol has shown a greater ability to predict progression to ACLF. These findings highlight the role of androgen-estrogen imbalance in the prognosis and clinical decompensation of liver disease^28^.

Also, Sun et al. indicated that FT has an inverse correlation with the Fibrosis Index - 4 (FIB-4 index), a noninvasive marker of hepatic fibrosis, whereas SHBG demonstrated a statistically significant positive association. These findings suggest that biologically active testosterone may exert a protective effect against fibrosis, possibly mediated by its anti-inflammatory actions, while increased SHBG levels may reflect lower hepatic fat accumulation and greater progression toward fibrosis. This interpretation is consistent with previous evidence linking elevated levels of inflammatory cytokines to the progression of MASLD from simple steatosis to steatohepatitis and fibrosis^27^.

Overall, the reviewed literature indicates that gonadal dysfunction in men with LC is not only a consequence of hepatic injury but also a predictor of poorer prognosis and increased mortality. Recognizing this association may help guide more individualized management strategies, including routine hormonal evaluation and the potential use of testosterone replacement therapy under careful monitoring. However, randomized clinical trials with larger samples and longer follow-up are still required to determine the true impact of hormonal replacement on survival and quality of life^21^ ^,^ ^23^.

With these considerations, the bundle developed in this study was primarily designed to evaluate gonadal function in men with LC, providing clinical support to healthcare professionals involved in the management of these patients, particularly in pre- and post-LTx settings. This approach is justified by the high prevalence of hypogonadism among men with cirrhotic and by the negative impact of this condition on metabolism, body composition, sexual function, bone mineral density­, and quality of life^3^ ^,^ ^36^ ^,^ ^37^.

In this context, the validation of instruments with clinical applicability is of particular relevance. The validation of clinical bundles by healthcare specialists represents a fundamental step in the development of tools intended for medical practice. In the present study, the characterization of the expert panel revealed a group of professionals with substantial clinical and academic experience, predominantly composed of specialists in hepatology and gastroenterology, many of whom are directly involved in LTx^12^.

The content validation results demonstrated high theoretical quality of the bundle, with global and domain-specific indices exceeding the recommended cutoff point, indicating consensus among the experts regarding its relevance, clarity, and applicability. The IA-I also indicated good reliability among the evaluators, reinforcing the consistency and stability of the assessments across specialists^16^.

From a theoretical perspective, the Objective domain stood out for accurately reflecting the main purpose of the instrument: to assist healthcare professionals in the assessment of gonadal function in patients with LC. This diagnostic phase is often underestimated in clinical practice, despite evidence indicating that up to 80% of men with advanced LC present reduced TT levels^38^.

In the Structure and Presentation domain, the content validity index of 98.2% indicates that the material was evaluated as scientifically appropriate, objective, and compatible with a specialized medical audience. Clarity of language and logical organization of the material are essential to ensure the applicability of technologies designed to support clinical decision-making^39^. The recommendation to adapt the design for smartphone visualization is particularly relevant, as mobile access currently represents one of the main forms of medical consultation at the bedside, facilitating continuous use and the broad dissemination of digital clinical protocols^40^.

The Relevance domain, which achieved the maximum score (100%), confirms that the bundle encompasses key elements of patient care and has potential applicability across different clinical settings. This evaluation is supported by evidence indicating that structured and interactive tools, when properly validated, enhance healthcare professionals’ adherence to better clinical practices and promote safer and more consistent care outcomes. 

The experts’ suggestions further reinforced the validation of the instrument by proposing adjustments that enhance its precision, interactivity, and clinical applicability, such as the use of flowcharts, clear definitions of clinical management strategies, and user-friendly digital interfaces. The integration of hormonal assessment into a multidisciplinary approach, involving endocrinology, nutrition, and physical exercise, is supported by evidence demonstrating metabolic improvement, reduction of systemic inflammation, and functional optimization in patients with LC and hypogonadism^41^.

From a therapeutic perspective, recent reviews and clinical trials emphasize that the use of testosterone therapy in patients with LC remains controversial. While some studies report benefits in body composition and bone mineral density, others highlight potential risks, including hepatotoxicity and progression of hepatocellular carcinoma, thereby requiring careful evaluation before clinical indication^38^.

## CONCLUSION

The development of the bundle highlighted the shortage of studies focused on the systematic evaluation of gonadal function in patients with LC, as well as the limited number of studies addressing associated endocrinological manifestations, especially hypogonadism and its therapeutic options. Furthermore, a significant gap in information regarding the clinical management of hypogonadism exists, both before and after LTx, exacerbated by the absence of longitudinal follow-up studies. In this context, the development and validation of the bundle represent an advance by offering a structured tool, based on evidence and expert consensus, capable of organizing the clinical and laboratory evaluation of gonadal function in men with LC. Future studies, with greater methodological robustness, may contribute to the standardization of requested tests and therapeutic approaches according to the clinical profile of the patients. Thus, the validated bundle assumes a strategic role in strengthening integrated and evidence-based care models, assisting individualized clinical judgment, promoting the standardization of practices, and reducing iatrogenic risks, with a potential positive impact on the quality of care provided to men with LC.

## Data Availability

The research data are presented within the article itself (available in Tables 1, 2, 3, 4 and Figure 1 and 3)
